# Readiness for digital transformation of higher education in the Covid-19 context: The dataset of Vietnam's students

**DOI:** 10.1016/j.dib.2021.107482

**Published:** 2021-10-14

**Authors:** Huyen Pham, Que-Nhi Tran, Gia-Long La, Ha-My Doan, Tien-Duc Vu

**Affiliations:** Marketing Faculty, National Economics University, Hanoi 100000, Viet Nam

**Keywords:** Digital transformation, Covid19, Awareness, Attitude, Self-study, TAM model

## Abstract

With the development of digital technology, Vietnam's education has been undergoing significant changes. This is considered one of the eight important fields of the National Digital Transformation, so it needs to take advantage of opportunities to be able to train high-quality human resources according to international standards. Beside, the Covid-19 pandemic has quickly put pressure on the previously predicted trends in education such as the “Future University”. This paper shows the data of an investigation on the factors affecting the readiness of Vietnamese students for digital transformation in the above context. The data is built based on the TAM model and sociological investigation method to collect multidimensional information from many perspectives of different individuals to have a basis for assessing the level of influence. The survey includes the main questions corresponding to the independent variables in the model: Self-study ability, Attitude, Perceived Usefulness, Perceived Ease of Use, and Covid-19. The authors distributed the questionnaire online and collected 913 valid responses.

## Specifications Table

**Table:** 

Subject	Social Science, Education, Digital Transformation
Specific subject area	Online learning, Distance learning, Digital Educational Transformation, Learning Method, Students’ Readiness
Type of data	Table Figure Data Excel
How data were acquired	Survey Questionnaire. All valid samples were processed with SPSS 26.0 and SPSS AMOS 20 software to conduct Cronbach's Alpha, EFA, CFA, SEM analysis.
Data format	Raw Analyzed
Parameters for data collection	The authors selected a convenient non-probability sampling method for students at universities who are living and working across the country, affected by Covid-19.
Description of data collection	Primary data sources were collected from distributing online questionnaires (Google Form) through social networks to students from January to February 2021. The authors distributed the questionnaire and collected 979 answer sheets. During the survey, there were 66 invalid votes because of choosing more answers than prescribed, answering not enough questions, and answering all the same answers. After cleaning data, there are 913 valid votes left.
Data source location	Authors’ survey, with sample of 913 students from universities Region: Asia Country: Vietnam Latitude and longitude: 16°00′N 108°00′E
Data accessibility	Data with the article

## Value of the Data

• The data is collected in the context of Vietnamese, under higher education moving towards digital transformation and being pressured by three waves of the Covid19 pandemic to assess students' readiness for digital transformation in learning and being affected by Covid-19. From that, propose solutions to improve the quality of teaching at universities and orient the development of a new educational model in the current context.
• The data is based on Davis's theory of technology acceptance model in technology and education research [1] because it has the excellent explanatory ability, with 86% of previous studies using the TAM model to build the research.
• The data is useful for the education sector managers and governments in drafting supportive policies for the University's online education model in the long run. Specifically, universities and colleges can benefit from assistance in developing appropriate policies to increase the quality of teaching and learning. Next, for lecturers - the subject of the activities of teaching and imparting knowledge, skills, experiences, this data may help them grasp the motivations and attitudes of students, thereby suggesting appropriate methods to support students.
• These data can be used and further developed from research related to higher education, digital transformation in learning and teaching for developing countries like Vietnam in the context of IR 4.0, and the impact of the Covid-19 pandemic. From there, our research will be completed in more detail and objective from many countries' perspectives in the future.

## 1 Data Description

The outbreak of the Covid-19 pandemic has put pressure on the education industry around the world. Until now, it is clear that the digital transformation truly brought innovation and solutions to the education industry. In response to the Covid-19 pandemic outbreak, the Ministry of Education and Training in Vietnam issued consecutively Official Letter 795/BGDT [2], and 988/BGDĐT-GDDH [3], directing the deployment of online training methods in educational institutions. Simultaneously, it is necessary to take advantage of IR 4.0 to close the space and time gap, bringing Vietnamese education closer to the quality of international education. For that reason, this paper presents the dataset of a survey on “COVID-19 and Factors Affecting Students' Readiness for Digital Transformation: Data of Vietnam”. The dataset included three major groups of variables: (1) Individual demographics, including gender, grade level, majors, tuition, and living region; (2) Factors affecting students’ readiness level, including perceived usefulness, perceived ease of use, self-study ability, and Covid-19; (3) Students’ perspective about the necessity of Digital Educational Transformation. There was a total of 979 answers to our survey, but only 913 responses were qualified. The questionnaire and collected data are provided in supplementary documents.

All demographic characteristic that authors supposed that can be affects to the students'' readiness level for digital transformation are illustrated in the Table 1.

**Table 1:** Respondents’ characteristics.

Number	Demographic		Frequency	Proportion (%)
1	Gender	Male	282	30,90%
		Female	631	69,10%

2	Grade level	First-year	240	26,30%

		Second-year	355	38,90%

		Third-year	239	26,20%

Fourth-year	46	5%

Above 4th year	33	3,60%

3	Majors	Educational science and teacher training	24	2,60%

		Humanities, Social Sciences and Behavior, Journalism and Defense Security	174	19,10%

		Business and Management	497	54,40%

		Law	52	5,70%

Life Science and Nature Science	9	1%

Statistics, Engineering and Computer & IT	61	6,70%

Health	80	8,80%

Art	16	1,80%

4	Tuition (per year)	From 0 to 15 million VND	278	30,40%

		From over 15 to 30 million VND	253	27,70%

		From over 30 to 50 million VND	199	21,80%

From over 50 to 100 million VND	119	13%

From over 100 to 300 million VND	37	4,10%

Above 300 million VND	27	3%

5	Region	Suburban	716	78,40%

Urban	197	21,60%

Table 2 demonstrates descriptive statistic the level of agreement to statements represents for factors that affecting students’ readiness for transformation into digital learning. They are including PU (perceived usefulness), PEOU (perceived ease of use), ATT (Attitute to online learning), SSA (self-study ability), COVID (Covid-19) and CHANGE (change to online learning).

**Table 2:** Descriptive results of students’ responses to the survey.

Variables	Items	N	Range	Minimum	Maximum	Mean	S.D	Variance
PU	The online learning system helps me absorb knowledge more effectively	913	4	1	5	3,36	1,005	1,009

	The online learning system helps me improve my academic results	913	4	1	5	3,73	0,927	0,859

The online learning system makes me more proactive in learning	913	4	1	5	3,81	0,921	0,847

PEOU	I suppose that it is easy for me to learn how to use the online learning system	913	4	1	5	3,9	0,884	0,781

	I suppose that the online learning system very easy to use	913	4	1	5	3,9	0,891	0,794

I believe that it is easy for me to competently use online learning systems	913	4	1	5	3,99	0,889	0,791

ATT	I suppose that it is necessary to use an online learning system	913	4	1	5	3,82	0,888	0,789

	I support the use of an online learning system	913	4	1	5	3,77	0,958	0,919

I suppose that using online learning is a good idea	913	4	1	5	3,8	0,967	0,934

I feel very excited when using an online learning system	913	4	1	5	3,47	1,082	1,17

SSA	I always actively interact with the lecturers during class	913	4	1	5	3,36	1,005	1,009

	I always actively participate in learning activities and do group exercises with my teammates	913	4	1	5	3,73	0,927	0,859

I always proactively arrange my own schedule	913	4	1	5	3,81	0,921	0,847

COVID	Covid-19 has helped me approach digital transformation in learning	913	4	1	5	4,03	0,889	0,791

	Covid-19 has helped me adapt to the shift in learning methods	913	4	1	5	4,02	0,896	0,802

Covid-19 has helped me feel excited about the new learning method	913	4	1	5	3,52	1,049	1,101

Covid-19 has helped me more proactive and self-disciplined in studying	913	4	1	5	3,5	1,09	1,189

CHANGE	I have been ready to acquire knowledge more proactively	913	4	1	5	4	0,856	0,732

	I have proactively absorbed knowledge through digital platforms	913	4	1	5	3,96	0,826	0,682

	I have proactively interacted with lecturers through digital platforms	913	4	1	5	3,69	0,947	0,897

I have proactively searched for learning materials through digital platforms	913	4	1	5	3,99	0,865	0,749

I have proficiently used digital platforms for learning and discussion	913	4	1	5	3,9	0,881	0,776

The reliability of above scales is checked with Cronbach's Alpha, presented in the Table 3.

**Table 3:** Results of verifying the reliability of the scale.

Factors	Cronbach's Alpha coefficient	Cronbach's Alpha if item deleted	Number of variables removed
Perceivable Usefulness (PU)	0,824	0,706–0,826	0/3
Perceivable Ease of Use (PEOU)	0,887	0,804–0,865	0/3
Attitude (ATT)	0,894	0,846–0,886	0/4
Self-study ability (SSA)	0,788	0,653–0,756	0/3
Readiness for digital transformation in learning (CHANGE)	0,912	0,882–0,910	0/5

To discover the factor structure of measurement scales and to examine its internal reliability, the Exploratory Factor Analysis has been use and the Table 4 shows the results.

**Table 4:** The results of exploratory factor analysis (EFA).

	Factor					
Pattern Matrixa	1	2	3	4	5	6
PEOU2	0,911					
PEOU1	0,821					
PEOU3	0,814					
PU1		0,903				
PU2		0,809				
PU3		0,627				
CHANGE2			0,908			
CHANGE4			0,862			
CHANGE5			0,79			
CHANGE1			0,776			
CHANGE3			0,657			
ATT2				0,911		
ATT4				0,872		
ATT3				0,859		
ATT1				0,643		
COVID2					0,959	
COVID1					0,805	
COVID3					0,494	
COVID4					0,417	
SSA2						0,813
SSA1						0,734
SSA3						0,617
Extraction Variance	3,018	1,061	7,627	1,205	0,863	0,735
Eigenvalues	3,326	1,365	7,963	1,540	1,203	1,072

**Fig. 1:**
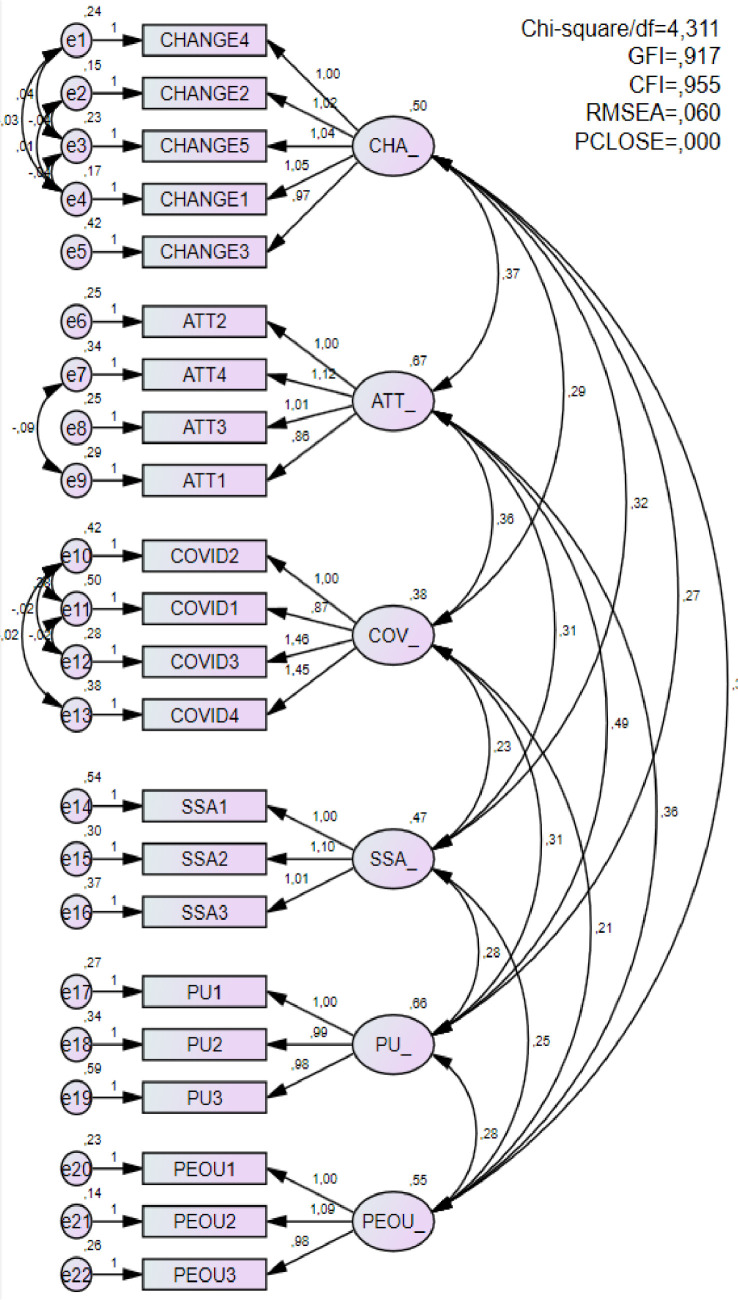
Confirmatory factor analysis.

Based on EFA results, Confirmatory Factor Analysis has been applied for verifying the factor structure of a set of observed variables, as Fig. 1 illustrated.

**Fig. 2:**
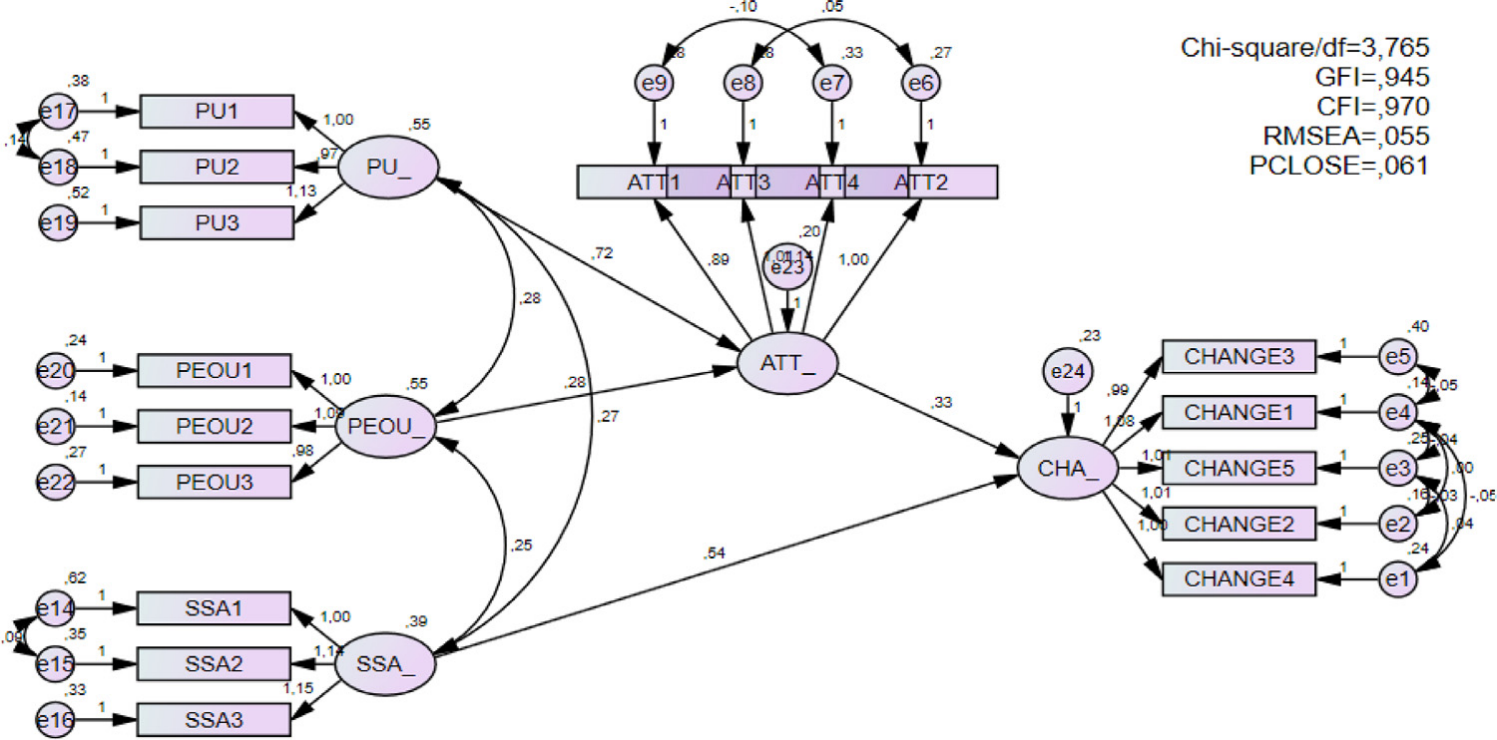
SEM results without control variables.

**Fig. 3:**
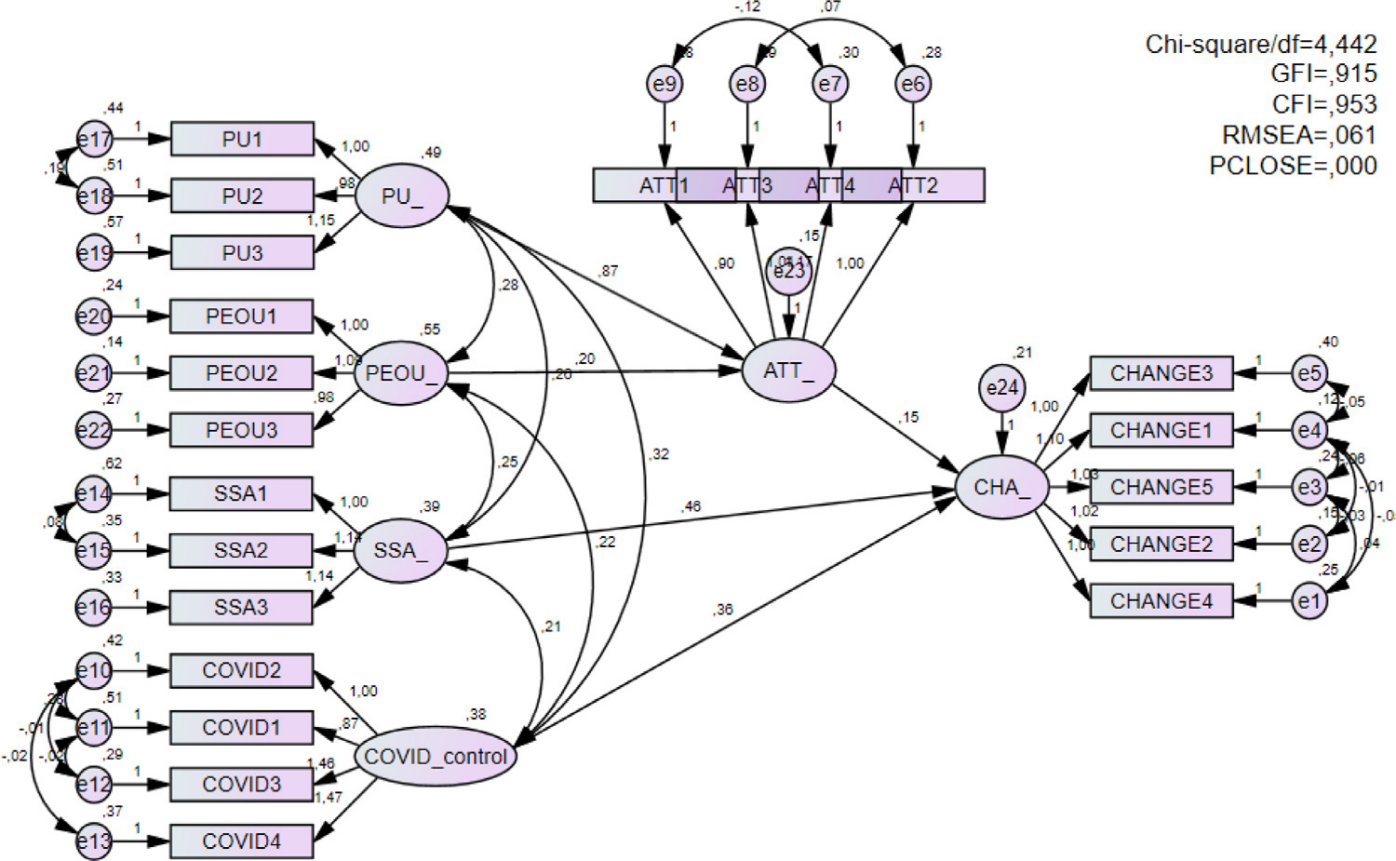
SEM results with control variable of COVID-19.

The results of testing the research model with/without taking into account the Covid19 as a moderator factor to the relationship between variables considered as factors affecting the readiness of Vietnamese students for digital transformation, are showed at Tables 5 –7 and Figs. 2 & 3.

**Table 5:** Summary of SEM without control variables results.

Hypothesis (Hs)		Regression weight	P-Value (Sig)	Result
H1a	ATT <— PU	0,725	0,000	Accepted
H1b	ATT <— PEOU	0,284	0,000	Accepted
H2	CHA <— ATT	0,329	0,000	Accepted
H3	CHA <— SSA	0,539	0,000	Accepted

**Table 6:** Summary of SEM with control variables Covid-19 results.

Hypothesis (Hs)		Regression weight	*P*-Value (Sig)	Result
H1a	ATT <— PU	0,725	0,000	Accepted
H1b	ATT <— PEOU	0,284	0,000	Accepted
H2	CHA <— ATT	0,329	0,000	Accepted
H3	CHA <— SSA	0,539	0,000	Accepted
H4	CHA <— COVID_ctrl	0,363	0,000	Accepted

**Table 7:** Perspectives on digital transformation in higher education.

	Frequency	Percent
Should not	217	23,8%
Should	696	76,2%
Total	913	100%

## 2 Experimental Design, Materials and Methods

### 2.1 Research model

**Fig. 4:**
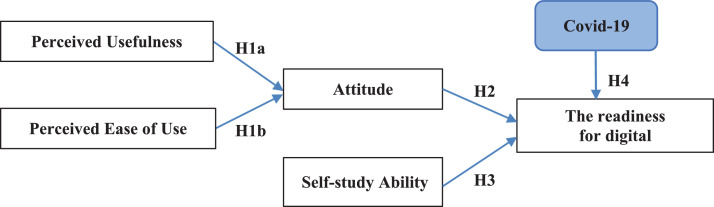
Proposed research model.

The survey was conducted based on the research model using the theory of technology acceptance model (TAM) because it is the most popular theory in the study of technology and education. King and He [4], Sumak et al. [5], Abdullah and Ward [6], and Al-Qaysi [7] argue that TAM is suitable to build a research model on students' technology use behavior. From the above reasons, combined with the results of in-depth interviews with survey subjects, the authors propose the research model, is shown in the Fig. 4.

### 2.2 Constructing and questionnaire processing

Based on theory and research overview, the authors built the research model and draft scales. This scale system was calibrated based on in-depth interviews with lecturers and students of various disciplines and training programs, from different universities and majors. Thereby, the authors have ensured that the scale was suitable for this context. On that basis, the authors designed the questionnaire and conducted a preliminary quantitative survey with 53 responses to check the suitability of the factors and scales for the proposed research model through systematic analysis of Cronbach's Alpha reliability number. After that, the completed questionnaire for a large-scale official quantitative survey with students currently studying at universities and colleges in Vietnam.

The research team uses a 5-point Likert scale; responses are obtained in each question by choosing the appropriate level from 1 to 5 points: (1) Totally disagree; (2) Disagree; (3) Normal; (4) Agree; (5) Totally agree.

### 2.3 Data collected and analyzing processed

The authors distributed the questionnaire and collected 979 responses. After cleaning, there are 913 valid responses left. During the survey, there were 66 invalid responses because of choosing more answers than prescribed, answering not enough questions, and answering all the same answers. All valid samples will be processed with SPSS 26.0 and SPSS AMOS 20 software to conduct reliability analysis, exploratory factor analysis, confirmatory factor analysis, and tissue analysis. SEM structural modeling and hypothesis testing.

In Table 4 the group also mentioned the EFA results performed by the method "Principal Axis Factoring" and the “Promax” rotation, specifically, there are 6 factors extracted from 22 observed variables. Eligible extraction variances were all > 50%. The results of CFA (Fig. 1) show that the scales of the independent variables have been grouped into the following groups: "Perceived usefulness" (PU), "Perceived ease of use" (PEOU), "Attitude" (ATT), “Self-study ability” (SSA).

After performing EFA and CFA analysis to select data and scales, the team used SEM model analysis technique through SPSS 26.0 and AMOS 20.0. The Structural Equation Model was performed by the authors twice with the control variable (Covid-19) for the purpose of comparing the impact level and multidimensional relationship of the independent variables (PU, PEOU, ATT, SSA) on the dependent variable (CHA).

The results of the SEM model without the appearance of the Covid-19 control variable are shown in Fig. 2 and Table 5 above. Table 5 summarizing the SEM results, which was presented in the Data Description section shows the relationship between the independent and dependent variables. As a control variable in the SEM model, COVID-19 also contributes to the dependent variable "Readiness for digital transformation" (CHA), and at the same time makes the relationships between factors might change. The obtained results are shown in Fig. 3 and Table 6.

Beside, the survey was built during the Covid-19 pandemic, the authors also collected students' opinions on whether or not to move toward digital transformation in higher education. This problem has also been summarized in Table 7.

## Ethics Statement

The authors ensured that the dataset is built on the voluntary spirit of the respondents. All of the respondents’ personal information is used for research purposes and will be handled in accordance with the principle of anonymity.

## CRediT authorship contribution statement

**Huyen Pham:** Conceptualization, Methodology, Project administration. **Que-Nhi Tran:** Formal analysis, Writing – original draft, Supervision. **Gia-Long La:** Data curation, Writing – original draft, Visualization. **Ha-My Doan:** Validation, Writing – review & editing. **Tien-Duc Vu:** Resources, Writing – review & editing.

## Declaration of Competing Interest

The authors declare that they have no known competing financial interests or personal relationships which have or could be perceived to have influenced the work reported in this article. We ascertain that the material presented in this manuscript will not infringe upon any statutory copyright and that the manuscript will not be submitted elsewhere while under Data in Brief review.

## Appendix Supplementary materials

Image, application 1

Image, application 2
