# Supplementary material for: Readiness for digital transformation of higher education in the Covid-19 context: The dataset of Vietnam's students
Source: Data Brief. 2021 Oct 14;39:107482. doi: 10.1016/j.dib.2021.107482 (PMC8529093; doi:10.1016/j.dib.2021.107482)
Supplement: Supplementary file 1 [file mmc1.pdf]

No :

STUDENT SURVEY

IR 4.0, COVID19 AND STUDENT'S LEARNING METHOD

Dear respondents!

Industry 4.0, digital transformation, and especially the Covid19 pandemic have put pressure on all of us to change the way we live, work and study. In the field of education, digital transformation has been affecting the learning behavior of learners at all levels, especially at universities. However, are those changes really useful? How can students acquire knowledge and practice required skills more effectively in the current context?

We look forward to receiving your comments by answering the questions below or ticking the answer option that suits your opinion. All information provided will be treated anonymously and collected to suggest solutions to enhance teaching and learning efficiency in the context of digital transformation! Many thanks!

I. ASSESSMENT OF STUDENT'S READINESS FOR DIGITAL TRANSFORMATION

How would you evaluate the following statements? *For each statement, choose your level of agreement on the following scale: (1) Very Disagree; (2) Disagree; (3) Normal; (4) Agree; (5) Very Agree.*

**1. Perceived Usefulness**					
The online learning system helps me absorb knowledge more effectively	1	2	3	4	5
The online learning system helps me improve my academic results	1	2	3	4	5
The online learning system makes me more proactive in learning	1	2	3	4	5
**3. Attitude**					
I suppose that it is necessary to use an online learning system	1	2	3	4	5
I support the use of an online learning system	1	2	3	4	5
I suppose that using online learning is a good idea	1	2	3	4	5
I feel very excited when using an online learning system	1	2	3	4	5
**5. Covid 19**					
Covid 19 has helped me approach digital transformation in learning	1	2	3	4	5
Covid 19 has helped me adapt to the shift in learning methods	1	2	3	4	5
Covid 19 has helped me feel excited about the new learning method	1	2	3	4	5
Covid 19 has helped me more proactive and self-disciplined in studying	1	2	3	4	5

**2. Perceived Ease of Use**					
I suppose that it is easy for me to learn how to use the online learning system	1	2	3	4	5
I suppose that the online learning system very easy to use	1	2	3	4	5
I believe that it is easy for me to competently use online learning systems	1	2	3	4	5
**4. Self-study ability**					
I always actively interact with the lecturers during class	1	2	3	4	5
I always actively participate in learning activities and do group exercises	1	2	3	4	5
I always actively arrange my own schedule	1	2	3	4	5
**6. Student's readiness for digital transformation**					
I have been ready to acquire knowledge more proactively	1	2	3	4	5
I have proactively absorbed knowledge through digital platforms	1	2	3	4	5
I have proactively interacted with lecturers through digital platforms	1	2	3	4	5
I have proactively searched for learning materials through digital platforms	1	2	3	4	5
I have proficiently used digital platforms for learning and discussion	1	2	3	4	5

7. Should higher education change towards digital transformation? ▢ Should ▢ Should not

II. PERSONAL INFORMATION: (All your personal information will be handled in accordance with the principle of anonymity)

8. Sex: ▢ Male ▢ Female ▢ Other

9. Academic Year ▢ First Year ▢ Second Year ▢ Third Year ▢ Fourth Year ▢ Above the fourth year

10. Majors: ……………………………………………

11. Tuition (VND per year):

❒From 0-15 million VND ❒From over 15-30 million VND ❒From over 30-50 million VND

❒From over 50-100 million VND ❒From over 100-300 million VND ❒More than 300 million VND

12. Living Area: ❒ Urban area ❒ Suburban

THANK YOU FOR COMPLETING OUR SURVEY!
